# Disposition Index in Active Acromegaly

**DOI:** 10.3389/fendo.2019.00637

**Published:** 2019-09-18

**Authors:** Dan Alexandru Niculescu, Roxana Dusceac, Andra Caragheorgheopol, Nicoleta Popescu, Catalina Poiana

**Affiliations:** ^1^Department of Endocrinology, Carol Davila University of Medicine and Pharmacy, Bucharest, Romania; ^2^Research Laboratory, C. I. Parhon National Institute of Endocrinology, Bucharest, Romania; ^3^Biochemistry Department, C. I. Parhon National Institute of Endocrinology, Bucharest, Romania

**Keywords:** acromegaly, disposition index, insulin sensitivity, insulin secretion, impaired glucose tolerance

## Abstract

**Background:** The relative contribution of reduced insulin sensitivity (S_i_) or insulin secretion to impaired fasting glucose (IFG) or diabetes mellitus (DM) has not been clarified in active acromegaly. An intravenous glucose tolerance test (IVGTT) was never used for the calculation of S_i_, acute insulin response (AIR_g_), and disposition index (DI) in this population. Our aim was to assess S_i_, AIR_g_ and DI using an IVGTT in acromegaly with normal (NGT) and abnormal glucose tolerance.

**Methods:** We performed an IVGTT in 13 patients (8 NGT, 2 IFG, and 3 DM; 5 males, age 47.9 ± 11 years, body mass index 29.7 ± 4.1 kg/m^2^) with active acromegaly (insulin-like growth factor-1 4.1 ± 1.3 × upper limit of normal, basal GH 29.1 ± 25 ng/mL) and 3 healthy controls (2 males, age 39 ± 3 years, body mass index 23 ± 5 kg/m^2^). No patient had any growth hormone- or glucose-lowering medication.

**Results:** NGT patients had significantly lower S_i_ than healthy controls but higher AIR_g_. Hyperglycemic and normoglycemic patients had similar S_i_. DM patients had severely diminished AIR_g_ (5–260 pmol × min/L) while IFG patients maintained their insulin secretion (3,862 and 912 pmol × min/L). Patients with abnormal glucose tolerance (IFG + DM) had a significantly lower DI (353 ± 350) than both NGT patients (3,685 ± 2,544) and healthy controls (5,837 ± 1,894; *p* < 0.01 for ANOVA).

**Conclusions:** Disposition index suggests that although reduced insulin sensitivity is characteristic of active acromegaly it is the impaired insulin secretion that mainly drives glucose intolerance. The clinical utility of DI in predicting DM in acromegaly must be further investigated.

## Introduction

Impaired glucose metabolism is a constant feature of active acromegaly (1). As a result, impaired glucose tolerance (IGT) or diabetes mellitus (DM) are frequent with rates varying from 25% (2, 3) to 12–37% (4), respectively.

Reduction of insulin sensitivity (S_i_) has been widely demonstrated in active acromegaly. Patients with acromegaly and normal glucose tolerance (NGT) have a decreased S_i_ compared to controls in both clamp (5) and basal indices (6, 7) studies. However, most studies reported a similar S_i_ in patients with IGT or normogycemia (3, 8). Although growth hormone (GH) is considered responsible for this effect, S_i_ correlates inversely better with serum insulin-like growth factor-1 (IGF-1) than GH (3, 9). Complete (8) or partial (10) control of acromegaly by surgery leads to improvement of S_i_ in all patients but restoration of NGT is variable (8).

Reduced insulin secretion is also involved in the IGT of patients with acromegaly. Insulin secretion, crude or adjusted for S_i_, is lower in patients with IGT compared with normoglycemic ones (3, 8, 11). In patients with abnormal glucose tolerance due to impaired S_i_, successful pituitary surgery restores normoglycemia in those with preserved beta cell function but not in those with abnormal insulin secretion (8). This is supported by the clinical observation that some patients with severely impaired S_i_ never develop hyperglycemia. However, the number of studies addressing insulin secretion in acromegaly is significantly lower than those assessing S_i_ due to methodological issues.

Disposition index (DI), the product of S_i_ and the acute insulin response to glucose (AIR_g_), the parameter used for the estimation of insulin secretion, is an independent predictor of DM (12). In addition, in patients with IGT, DI can help identifying the involved pathogenic and compensatory mechanisms (13–15). The main difficulty in the widespread use of DI is the need of an intravenous glucose tolerance test (IVGTT) for the calculation of S_i_ and AIR_g_. Although IVGTT is technically demanding and not suitable for routine clinical practice it is more accurate than its oral counterpart (14).

To our knowledge, no study assessed the DI and its relationship with glucose tolerance in patients with active acromegaly. Also, IVGTT was never used for calculation of S_i_, AIR_g_, and DI in this population. Our aim was to assess insulin sensitivity and insulin secretion in active acromegaly using an IVGTT and to compare DI in healthy adults and acromegalic patients with normal and abnormal glucose tolerance status.

## Materials and Methods

### Subjects

We assessed 13 patients with active acromegaly and 3 healthy controls. Of the 13 patients, 9 (69%) had newly diagnosed acromegaly with no previous treatment, 3 (23%) had previous unsuccessful sphenoidal surgery and 1 (8%) had pituitary high-voltage radiotherapy. No patient was on any medical treatment for acromegaly. Active acromegaly was confirmed based on unsuppressed serum growth hormone (GH>1 ng/mL) during an oral glucose tolerance test (OGTT) in patients with NGT/IFG or on a 5-point mean serum GH>2.5 ng/mL in diabetic patients. IGF-1 was increased in all patients. Pituitary function (except the GH axis) was normal in all patients.

Eight (61.5%) patients had NGT, 2 (15.4%) had impaired fasting glucose (IFG) and 3 (23.1%) had DM. None of the 5 patients with abnormal glucose tolerance had any glucose-lowering medication at the moment of IVGTT but 2 were on diet. All 3 healthy controls had NGT and were free of any known medical conditions or medication. Glucose tolerance was diagnosed based on a 75-grams OGTT. Patients' and controls' characteristics can be found in Table 1.

**Table 1:** Patients' and controls' basal characteristics.

**Glucose tolerance**	**Acromegaly**			**Controls**	*p*-value^*^
	**NGT**	**IFG**	**DM**	**NGT**	
Male: Female	5:3	2:0	2:1	2:1	NA
Age (years)	45.6 ± 10	42/45	69/61/41	39 ± 3	0.13
BMI (kg/m^2^)	30 ± 3.5	29/37	23/25/33	23 ± 5	0.11
Weight (kg)	90 ± 14	98/112	75/62/95	74 ± 23	0.34
Glucose dose (g)	27 ± 4.2	29.4/33.6	22/19/33	22.2 ± 7	0.34
Basal GH (ng/mL)	9.4 (5.4, 42.4)	26.5/51	10/28/68	0.05 ± 0.01	<0.001
IGF-1 (x ULN)	3.8 (3.3, 4.7)	6.1/4.3	3.1/2.3/6.3	0.9 ± 0.08	<0.001
FBG (mmol/L)	5 ± 0.34	6.5/6.7	6.3/10.5/7	4.8 ± 0.36	0.59
120' BG (mmol/L)	5.5 ± 0.93	10.6/7.2	NA	4.6 ± 0.6	0.13

* *For controls vs. acromegaly with NGT*.

*Data are presented as mean ± SD except GH and IGF-1 (median [25, 75 percentile]. Data in IFG and DM groups are presented as individual values*.

*BMI, body mass index; DM, diabetes mellitus; FBG, fasting blood glucose; 120' BG, boold glucose 120 min after an oral glucose load; GH, growth hormone; IFG, imaired fasting glucose; IGF-1, insulin-like growth factor-1; NGT, normal glucose tolerance; ULN, upper limit of normal*.

All patients and healthy controls signed an informed consent approved by our Institution.

### IVGTT Protocol

The IVGTT was performed after an overnight fast. Two intravenous cannulas were inserted in the antecubital veins of both arms. Two 3 mL samples of blood were drawn 5 min apart for the measurement of serum glucose, insulin, GH and IGF-1. A bolus of glucose (0.3 g/kg of body weight in a 33% solution) was given within 60 s into the antecubital vein. Blood was sampled from the contralateral antecubital vein at 2, 3, 4, 5, 6, 8, 10, 15, 20, 25, 30, 40, 50, 60, 75, 90, 120, and 180 min after the end of glucose infusion for the assessment of the serum glucose and insulin levels.

### Biochemical Assays

Serum glucose was measured using an automated absorption photometry method (Cobas C501- ROCHE), measuring range 0.11–41.66 mmol/L.

Serum insulin was measured using an automated chemiluminiscent method (Access 2—Beckman Coulter), measuring range 0.21–2,100 pmol/L.

Growth hormone and IGF-1 were measured with an automated chemiluminescent analyzer (Liaison XL, Diasorin). Measuring ranges were 3–1,500 ng/ml for IGF-1, and 0.05–80 ng/ml for GH, respectively. GH assay is referenced to the WHO Second IS 98/574 for Somatotropin (22-kDa recombinant DNA-derived materials).

### Computation of Disposition Index

Insulin sensitivity (S_i_) was calculated using k_G_, the rate of glucose elimination from the blood, and AUC_ins_, the area under the curve of serum insulin between 0 and 75 min after glucose infusion. k_G_ was computed as the slope of the glucose elimination curve between 0 and 75 min using the following equation:

$$\begin{array}{l} f\left(t\right)=\text{a }e^{-kt} \end{array}$$

where a is a constant, t is the time after glucose infusion and *f (t)* is the serum glucose concentration. The AUC_ins_ between 0 and 75 min was computed using the linear trapezoid method. S_i_ was calculated as:

$$\begin{array}{l} S_{i}=\left(k_{G}\times \;10^{6}\right)÷AUC_{ins} \end{array}$$

and is measured in (L × 10^6^)/(pmol × min).

Insulin secretion was calculated as the AIR_g_, the ΔAUC_ins_ (above baseline) between 0 and 10 min after glucose infusion. AIR_g_ was computed using the linear trapezoid method and is expressed in pmol × min/L.

Glucose and insulin responses to glucose administration were divided into a first-phase response (0–10 min after the end of glucose administrations, above baseline) and a second-phase response (10–75 min after the end of glucose administration, above baseline).

Disposition index was calculated as the product of insulin sensitivity and insulin secretion:

$$\begin{array}{l} DI=S_{i}\times AIR_{g} \end{array}$$

### Statistics

A Kolmogorov-Smirnov test was performed for all data sets to check for normality. All data are expressed as mean ± standard deviation (SD) or as difference (95% confidence interval [CI 95%] for the difference) (data sets from populations with normal distribution) except basal GH and IGF-1 [median (25, 75 percentiles)]. Due to the low number of cases and wide variation data in IFG and DM groups are presented as individual values. For comparisons between two groups the Student's *t*-test was used. For paired comparisons inside the same group a paired Student's *t*-test was used. For comparisons between three groups we used one-way ANOVA. A *p*-value <0.05 was considered statistically significant. All statistical tests were carried out using MedCalc Statistical Software (version 14.8.1, MedCalc Software bvba, Ostend, Belgium). For graphics we used SigmaPlot Software (version 12.5, Systat Software inc, San Jose, CA).

## Results

### Glucose Curves

Healthy subjects had a very steep increase in serum glucose levels followed by a short relative plateau until 5 min after the glucose infusion. Afterwards, the glucose levels had an exponential decrease and reached the basal levels at 40 min [difference 0.46 (CI 95% −1.5 to 2.4) mmol/L; *p* = NS]. Serum glucose continued to decrease until 75 min to a level significantly lower than baseline [−1.05 (CI 95% −0.7 to −1.5) mmol/L; *p* < 0.01] and then recovered.

In patients with acromegaly and NGT the glucose peak was lower than in controls but the difference reached statistical significance only at 5 min [−2.78 (CI 95% −0.2 to −5.3) mmol/L; *p* = 0.01]. The exponential decrease was flattened, and the glucose levels reverted to baseline only at 60 min [difference 1.15 (CI 95% −0.08 to 2.4) mmol/L; *p* = 0.06].

**Figure 1:**
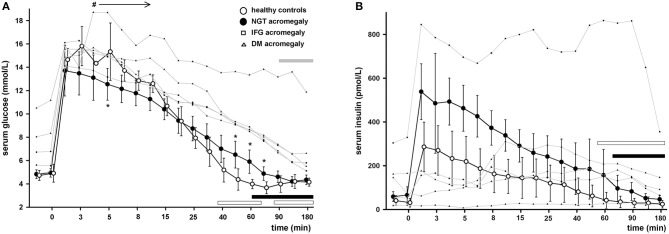
Serum glucose **(A)** and serum insulin **(B)** during IVGTT in healthy controls (empty circles) and patients with acromegaly and nomoglycemia (filled circles), IFG (small squares) or DM (small triangles). Data are presented as mean ± SD in controls and normoglycemic patients or as individual values in IFG/DM patients. *p < 0.05 NGT patients vs. controls, ^#^*p* < 0.05 IFG/DM vs. NGT patients. Horizontal bars denote time points when glucose or insulin levels were similar to baseline in healthy controls (empty bars), NGT patients (black bars) or IFG/DM patients (gray bars). Note that the time axis scale is not linear.

In patients with acromegaly and abnormal glucose tolerance (IFG or DM) the glucose levels were higher than in NGT patients starting with min 4 until the end of the test (min 180). The decrease of serum glucose was very slow, and the glucose levels reverted to baseline only at min 90 [difference 1.13 (CI 95% −0.7 to 2.9) mmol/L; *p* = NS]. The glucose curves in healthy subjects and patients can be found in Figure 1A.

**Figure 2:**
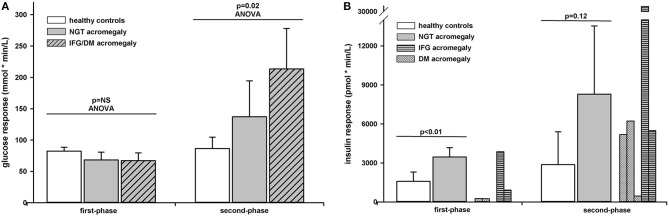
Glucose **(A)** and insulin **(B)** responses to intravenous glucose administration in healthy controls (white bars) and patients with acromegaly and NGT (gray bars) or IFG/DM (shaded bars). Data are presented as mean ± SD. The insulin responses are presented as individual data in patients with DM (dotted bars) or IFG (horizontally shaded bars).

The first-phase glucose response (0–10 min) was similar in the three groups (*p* = 0.18 ANOVA) (0–10 min). In pair comparisons it was 14.14 (CI 95% −3.2 to 14.1) mmol × min/L lower in NGT patients than in healthy controls but the difference did not reach significance (*p* = 0.09). The first-phase glucose response was also similar between patients with acromegaly with or without glucose intolerance [difference 1.13 (CI 95% −14.4 to 16.7] mmol × min/L; *p* = NS). The second-phase glucose response (10–75 min) differed significantly between the three groups (*p* = 0.02 ANOVA). In pair comparisons it was 50.6 (CI 95% −27.7 to 129.0) mmol × min/L higher in NGT patients with acromegaly than in healthy controls but the difference did not reach significance (*p* = 0.17). In patients with abnormal glucose tolerance the second-phase glucose response was 76.2 (CI 95% 0.8 to 151.6; *p* = 0.04) mmol × min/L higher than in acromegaly with NGT. The first- and second-phase glucose responses can be found in Figure 2A.

### Insulin Curves

Healthy subjects had also a very steep increase in serum insulin levels. They peaked at 2 min followed by a slow decrease. At 60 min the insulin levels were virtually to the same as the baseline [difference 10.7 (CI 95% −34.3 to 55.9) pmol/L; *p* = NS].

In patients with acromegaly and NGT the insulin levels followed a similar curve but were significantly higher than in controls between 2 and 90 min. They reverted to baseline only at 75 min [difference 29.9 (CI 95% −33.5 to 93.3) pmol/L; *p* = NS]. Patients with acromegaly and abnormal glucose tolerance had very different profiles ranging from almost no insulin release in response to increased glucose levels to very high insulin levels with no progressive decrease. Of the patients with preserved insulin secretion (2 with IFG and 2 with DM) 3 had a characteristic delayed response to glucose with no first phase insulin secretion and 1 had a vigorous response but with no gradual decrease. The insulin curves in healthy subjects and patients can be found in Figure 1B.

For first- and second-phase insulin response see the *Insulin secretion* section.

### Insulin Sensitivity

Healthy subjects had a higher basal insulin sensitivity than NGT patients as shown by HOMA-IR but the difference did not reach statistical significance (Table 2). Hyperglycemic patients had HOMA-IR indexes between 1.4 and 11.7.

**Table 2:** Insulin sensitivity, insulin secretion, and disposition index.

**Glucose tolerance**	**Acromegaly**			**Controls**	*p*-value^*^
	**NGT**	**IFG**	**DM**	**NGT**	
HOMA-IR	2.1 ± 1.4	11.7/2.3	8.7/2.4/1.4	1.01 ± 0.7	0.11
k_G_	0.016 ± 0.007	0.008/0.01	0.01/0.009/0.004	0.022 ± 0.001	<0.01
AUC_ins_0–75 (pmol^*^ min/L)	15, 448 ± 6, 299	57,838/10,394	17,570/11,742/1,747	7, 097 ± 4, 618	0.04
S_i_ [(10^6^ ^*^ L)/(pmol^*^ min)]	1.04 ± 0.63	0.13/0.96	0.56/0.76/2.28	5.01 ± 4.4	0.02
ΔAUC_ins_0–10 (pmol^*^ min/L)	3, 464 ± 712	3,862/912	260/258/5	1, 583 ± 723	0.02
Disposition index	3, 685 ± 2544	534/878	70/187/0.2	5, 837 ± 1, 894	0.22

* *For controls vs. acromegaly with NGT*.

*Data are presented as mean ± SD. Data in IFG and DM groups are presented as individual values*.

*AUC, area under curve; DM, diabetes mellitus; HOMA-IR, Homeostatic Model Assessment - Insulin Resistance; IFG, imaired fasting glucose; k_G_, the slope of the glucose elimination curve between 0 and 75 min; NGT, normal glucose tolerance; S_i_, insulin sensitivity*.

S_i_ was 3.9 (CI 95% 0.6 to 7.3) (L^*^10^6^)/(pmol × min); *p* = 0.02 higher in healthy controls than in NGT patients (Table 2). There was no difference in S_i_ between NGT and hyperglycemic patients. Both components of stimulated S_i_, k_G_, and AUC_ins_0-75, were different between healthy controls and NGT patients. Healthy controls showed a significantly higher k_G_ than NGT patients [difference 0.008 (CI 95% 0.004 to 0.012); *p* < 0.01]. Hyperglycemic patients had lower k_G_ than NGT patients [difference 0.005 (CI 95% 0.002 to 0.009); *p* < 0.01]. Also, healthy controls had significantly lower AUC_ins_0-75 than NGT patients [difference 9128 (CI 95% 46 to 18,211) pmol × min/L; *p* = 0.04). Hyperglycemic patients had extreme variations of this parameter, ranging from 1,747 pmol ^*^ min/L to 57,838 pmol × min/L (Table 2).

### Insulin Secretion

The first-phase insulin response (0–10 min) or AIR_g_ was 1881.5 (CI 95% 786 to 2,976) pmol × min/L; *p* < 0.01) higher in NGT patients than in healthy controls. As seen from the individual curves, in patients with acromegaly and abnormal glucose tolerance the first-phase insulin response varied widely from 5 to 3,862 pmol × min/L (Table 2). The second-phase insulin response (10–75 min) was 5,413 (CI 95% −1,909 to 12,736) pmol × min/L higher in NGT patients than in healthy controls but the difference did not reach significance (*p* = 0.12). The second-phase insulin response had also a wide variation in patients with abnormal glucose tolerance preventing a statistical approach. The first- and second-phase insulin responses can be found in Figure 2B.

### Disposition Index

**Figure 3:**
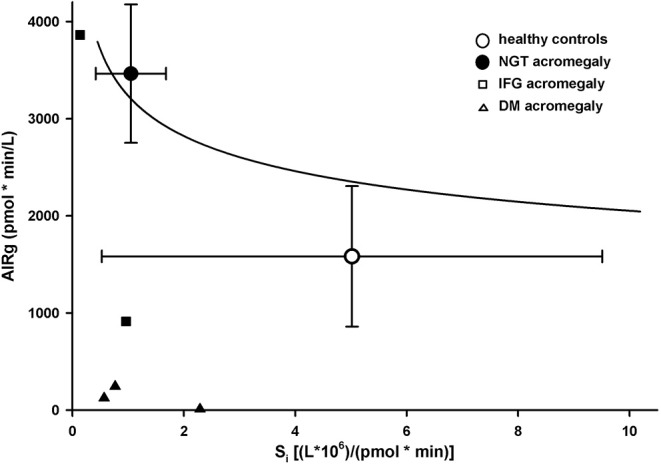
Disposition index in healthy controls (empty circles) and patients with acromegaly and nomoglycemia (filled circles), IFG (small squares), or DM (small triangles). Data are presented as mean ± SD. Solid line is the regression line for normoglycemic healthy controls and patients.

Patients with abnormal glucose tolerance (IFG + DM) had a significantly lower DI (353 ± 350) than both NGT patients (3,685 ± 2,544) and healthy controls (5,837 ± 1,894; *p* < 0.01 for ANOVA; Table 2). All hyperglycemic patients were below the DI curve for normoglycemic patients and controls (Figure 3).

### Correlation With Disease Activity

In patients with preserved insulin secretion (NGT and IFG) IGF-1 levels correlated with HOMA-IR (*r* = 0.62, *p* = 0.05) and S_i_ (*r* = −0.48, *p* = 0.15). Basal GH did not correlate with HOMA-IR (*r* = 0.03, *p* = 0.93) or S_i_ (*r* = −0.28, *p* = 0.4).

## Discussion

In this study we used an IVGTT protocol in patients with active acromegaly for calculation of S_i_, AIR_g_, and DI. These parameters were computed in both patients with normal and abnormal glucose tolerance and healthy controls. To our knowledge this is the first study to use IVGTT in acromegaly and to evaluate DI across categories of glucose intolerance.

We showed that NGT patients and healthy controls have significantly different serum glucose and insulin curves after intravenous glucose administration. Compared with healthy controls, NGT patients had a lower glucose peak, probably reflecting the higher distribution volume in acromegaly (16). However, the decrease of serum glucose is flattened, with higher levels than in controls starting with 25 min after the glucose infusion as a result of reduced S_i_. Patients with acromegaly and abnormal glucose tolerance had an even slower clearance of glucose from plasma. This feature is not surprising as patients with various grades of abnormal glucose tolerance but without acromegaly have a characteristic delayed clearance of glucose during an IVGTT (17).

Insulin levels were significantly higher in NGT patients reflecting the compensatory pancreatic response in the face of reduced S_i_ similar to other conditions of insulin resistance like obesity (18). Even more interesting were the individual insulin responses of the patients with abnormal glucose tolerance. Although they varied widely, preventing any statistical approach, it can still suggest the main pathogenic mechanism behind glucose intolerance and its evolution. The two IFG patients preserved their first-phase and second-phase insulin responses, similar to their non-acromegalic counterparts (17). However, the absolute insulin levels differed strongly from one another and from NGT patients (Figure 1B). It is tempting to suggest that the cure of acromegaly would reverse IFG in the first patient (vigorous insulin response, higher than in NGT acromegaly) but not in the second one (slight insulin response, lower than in NGT acromegaly). Two of the DM patients had a delayed insulin response with relative hyperinsulinism (vs. NGT acromegaly and healthy controls) toward the end of IVGTT, similar to non-acromegalic diabetic patients (19). The third DM patients had an almost absent insulin response.

Insulin sensitivity was significantly reduced in NGT patients, confirming previous studies on patients with active acromegaly. Our protocol uses an IVGTT-based calculation of S_i_, thus adding a new methodology to the existing ones: basal indices (6, 7) or euglycemic clamp (5). Interestingly, patients with abnormal glucose tolerance showed an important variation of S_i_ but not different from NGT patients. Given the low insulin secretion of these patients, this confirms the hypothesis that in active acromegaly, glucose intolerance is driven mainly by the inadequate insulin secretion in the face of reduced insulin sensitivity. It is tempting to suggest that the glucose intolerance of acromegaly follows the pattern of type 2 DM: increased GH levels play the role of obesity by reducing S_i_ (9) but only patients with diminished pancreatic function develop diabetes.

Although BMI was higher in NGT patients (30 ± 3.5 kg/m^2^) than in control subjects (23 ± 5 kg/m^2^) the difference was not statistically significant, possibly due to the low number of subjects. In the general population, excess fat and higher BMI impair S_i_ but it is unclear whether the higher BMI of acromegaly is due to increased fat tissue or to water retention and larger bones (16).

We were also able to show that DI was, as expected, significantly lower in IFG/DM patients than in NGT patients or healthy controls. DI was also lower in NGT patients than in healthy controls, but the difference was not statistically significant. Lower DI was shown to predict conversion to type 2 DM (12) in the general population. However, in patients with acromegaly the correlation between DI and the risk of DM might be not as clear as successful or partially successful treatment of acromegaly can dramatically improve S_i_ and consequently DI (8, 10). It would be of interest to see if NGT patients with lower DI are prone to develop IFG or DM later in life, even after successful treatment of acromegaly.

Another possible use of IVGTT and DI is to anticipate the effect of somatostatin analogs (SSA) on glucose metabolism. Although efficacious on GH and IGF-1 reduction SSA can have deleterious effects on glucose metabolism due to their direct inhibition of glucose release (20). Interestingly, SSA can improve or worsen glycemic control in different patients (21). Disposition index can show the relative contribution of S_i_ and insulin secretion as causes of glucose intolerance. In patients in whom the reduced S_i_ is the main cause behind glucose intolerance the reduction of GH/IGF-1 induced by the SSA would increase S_i_ more than the direct insulin inhibition with the result of improved glucose tolerance. In patients with a severely diminished insulin secretion SSA might worsen the glucose intolerance.

The main limitation of our study is the low number of subjects. This prevented the use of statistics in some areas of the study. Moreover, some differences did not reach statistical significance due to the low number of observations. The small number of subjects is mainly due to the complexity of IVGTT. Also, according to the protocol, none of the patients with abnormal glucose tolerance were on any glucose lowering medication and all patients with acromegaly were free of any medical treatment for acromegaly and had normal pituitary function. These stringent criteria further diminished the number of subjects. However, we were still able to demonstrate some important differences between controls and NGT acromegaly and between NGT and IFG/DM patients without any interfering effects of concomitant medication or abnormal pituitary function. Also, we showed that IVGTT and derived parameters can be successfully used in acromegaly to investigate the glucose metabolism. The risk of a type II error cannot be overlooked. For the main results of the study a potential type II error is most important for the lack of difference in the S_i_ between NGT and hyperglycemic patients. However, this was confirmed by other studies (3, 7).

The main advantage of this study is the use of an IVGTT to quantify S_i_ and, more importantly, AIR_g_. Many studies, with different approaches, confirmed the reduced S_i_ in active acromegaly (5–7). However, the IVGTT allowed us to measure insulin secretion and to show that glucose intolerance in active acromegaly is mostly dependent on insulin secretion and not on insulin sensitivity. Another advantage of our study is that patients with abnormal glucose tolerance were free of any glucose lowering medication. Also, most patients were newly diagnosed with acromegaly and had no current GH lowering treatment.

In conclusion, IVGTT can be successfully used for investigation of glucose metabolism in patients with acromegaly. Our data suggest that although GH excess reduces insulin sensitivity it is the impaired insulin secretion that drives glucose intolerance. The clinical utility of DI in predicting DM must be further investigated.

## Data Availability Statement

The datasets generated for this study are available on request to the corresponding author.

## Ethics Statement

The studies involving human participants were reviewed and approved by Ethics Committee, Carol Davila University of Medicine and Pharmacy. The patients/participants provided their written informed consent to participate in this study.

## Author Contributions

DN collected and analyzed the data, performed IVGTTs, wrote, reviewed and edited the manuscript. RD performed IVGTTs and reviewed the manuscript. AC performed the insulin, GH and IGF-1 measurements and reviewed the manuscript. NP performed the glucose measurements and reviewed the manuscript. CP analyzed the data, wrote and reviewed the manuscript.

### Conflict of Interest

The authors declare that the research was conducted in the absence of any commercial or financial relationships that could be construed as a potential conflict of interest.
